# Podalic Version

**Published:** 1918-12

**Authors:** P. W. Van Peyma

**Affiliations:** Buffalo, N. Y.


### Podalic Version.

P. W. VAN PEYMA, M. D., Buffalo, N. Y.

Of the various methods of practicing podalic version the writer's experience has been by far the greatest with what may be called the internal combined method, that is, the other hand co-operating over the abdomen. In a large experience, especially consulting and institutional, the writer has performed this operation many hundred times.

In the vast majority of cases where podalic version is indicated this method is decidedly the one of choice. The exclusively external method and the so-called Braxton Hick's method are comparatively valueless. The former frequently fails in execution or in the maintenance of the new presentation. The latter being practiced while the cervix is still only partially dilated leaving the further dilatation to be accomplished by the child is very sure to result in a dead child. The objection raised to the introduction of the hand into the uterus that it is very likely to cause infection is disproved by the experience of the writer. This subject is discussed under the head of practical asepsis. With very rare exceptions version should be followed by immediate extraction, and this especially in the interest of the child. Its accomplishment implies that the parturient canal is in condition to permit this with safety to the maternal parts and with safety to the child. In a multipara with a fully dilated cervix this condition generally obtains. In a primipara or in case of imperfect cervical dilatation the parturient canal must first be prepared.

Manual dilatation under an anaesthetic is the means generally to be employed. In cases where the dilatation will require considerable time various rubber dilators may be employed. The writer's usual practice is as follows: The external parts having been thoroughly cleaned, the vagina is irrigated with a one-half per cent solution of creoline. Both creoline and lysol have the valuable quality of being lubricant. Deep anaesthesia is generally demanded. The hand thoroughly covered with sterilized and two per cent carbolized vaseline is introduced into the vagina. The cervix is now carefully dilated by what is commonly known as Harris' method, that is one finger and the thumb, two fingers and the thumb and so on until the conically shaped hand passes readily through the cervix into the uterine cavity. The next step is to close the hand and by gradual traction withdraw the closed fist. Great care must be taken in the latter stages of this operation not to tear the cervix. The dilatation must not be hurried. The closed fist is next drawn through the vulvar orifice. In the case of primipara this also requires time, care and gentleness to guard against tears and abrasions. Whether the amount of dilatation thus accomplished will permit of rapid and safe delivery will depend upon the relative size of the foetal head and of the closed fist of the accoucheur. Additional dilatation can be readily obtained by placing the fingers of the other hand over the closed fist and slowly bringing this mass through the cervix and vagina.

With the membranes still intact and with full dilatation of the soft parts version and extraction in suitable cases can be readily done. Suitable cases are those where there is an indication for the operation, with no counter indication, and especially, no absolute disproportion between the child and the pelvis.

Cases of vertex or face presentation, where the head must be pressed aside before the hand can reach the foot within the cavity of the uterus, and in which later it must be pushed well up as the foot is brought down, present, other things equal, more difficulty than do transverse presentations where the feet are more accessible and where the head is not in the way. A small but vigorous hand is a great advantage to an obstetrician.

The novice is very apt to be impressed by the prominence of the lumbo sacral angle or promontory and is quite sure to conclude that the condition is abnormal—is one of shortened conjugate. The hand having passed this point further progress is generally easy. The introduction of the hand must be in the intervals between the pains always resting quiescently during pains. Very frequently the arms of the foetus are more accessible, more easily reached, than are the feet or legs, and are sometimes mistakenly brought down. In not a few cases the hand of the accoucheur must be introduced practically to the uterine fundus. The hand being well within the uterine cavity plenty of time should be taken to corroborate or disprove the previous diagnosis of position, and to determine the exact relation of the foetal parts. In cases where the membranes are intact one may often pass up between them and the uterine wall, finally going through them high up, perhaps after having distinctly located the various parts and decided where to grasp the proper foot. By this management often less amniotic fluid escapes and the version is insofar easier. The hand whose palm or surface naturally faces the foetal abdomen is generally the one to be introduced. The other hand co-operates over the mother's abdomen.

In all cases where the position is such that one foetal leg lies anterior to the other, the anterior leg must be brought down. This is a rule of the greatest importance. The chief reason for choosing the anterior leg is that traction upon the foetal leg cannot be far enough backwards to be in line with the axes of the upper pelvic planes but is necessarily forwards as well as downwards, with the result that if the posterior leg is brought down traction upon it brings it forward under the symphysis. This means that the foetal body is caused to rotate upon its long axis, and if as often happens, the arms go up, one or the other is quite sure to get behind the neck, and caught between it and the symphysis. This is a position from which it is very difficult to dislodge the arm, and the result is dangerous delay with very likely a broken humerus and clavicle and a dead child. Other valid reasons for choosing the anterior leg are that traction approximating the proper direction is easier, and again when the posterior leg is brought down the anterior hip is likely to catch on the symphysis. Bringing down both legs is very rarely necessary or even advisable.

A version, especially in cases of cephalic presentation, is not complete when the foot has only been brought down to just outside the cervix. The condition then is very likely to be one of the head and foot wedged in together; a condition of impaction which may be difficult to resolve. In all these cases it is of the greatest importance to work at both poles; that is while drawing down the leg to press upon the head. The writer has seen in consultation a number of cases where the attendants failed to deliver because failure to work at both poles had brought about a wedged impaction. In all the manipulations both hands, the internal and the external, work in unison. When the ankle is sufficiently low so that a fillet can be applied, traction should be made externally upon this with upward pressure upon the head and with the upward pressure by equivalent pressure downwards upon the uterine fundus. And especially in neglected cases the greatest care must be taken in upward pressure that it may not result in rupture of the uterus. The amount of force that is safe is a matter of good judgment acquired by large experience and an exact recognition of the actual state.

Version having been effected extraction follows. Pressure on the uterine fundus is of great importance. In all cases the bladder must be empty. The less the traction and the greater the pressure from above the less likely are the arms to go up and the head to become extended. The assistant exercising the outside pressure should stand high enough to make it effective. Frequently it is well that the patient who has perhaps been deeply anaesthetized should be allowed to recover sufficiently so that the uterine contractions and the bearing down efforts of the patient may be brought into play.

The leg which has not been brought down needs no attention. As soon as the body is born as far as the umbilicus a loop of the cord should be drawn down so as to give slack and guard against undue traction upon the umbilical attachment.

If the parts are well dilated and readily dilatable the final delivery is generally remarkably easy. In many of these cases the shoulders and the head almost fall out. In cases of delay at the pelvic brim firm pressure downwards towards the mother's back, not forwards towards the symphysis as is too often done, should be practiced. The assistant exercising this pressure should know upon which the occiput lies and his pressure should be somewhat towards that side of the pelvis so as to bring the wide biparietal diameter to that side of the promontory and thus avoid its engagement in the narrow conjugate. After the head has passed the brim and only then should the pressure be mostly upon the forehead or sinciput so as to insure good flexion. Two important advantages of version over high forceps are that the head enters by the smaller end of the wedge and that not being completely flexed it passes through the brim with the parietal diameter to one side of the narrow conjugate. Having entered the pelvic cavity flexion is an advantage and traction at this time should not be made before it has been obtained. This can very generally be aided by traction over the malar bones or with the fingers in the mouth. In the latter manipulation gentleness is requisite to guard against injury to the child.

When the mouth is beyond the perineum and respiration becomes possible the further delivery can be accomplished deliberately. The more complete has been the previous dilatation the less is the danger of perineal tears or of dangerous delay.

With good dilatation also, as has been said, there is rarely any difficulty in the delivery of the shoulders and head through the vulva. In many cases no particular manouver is required. In other cases the usual practice of raising the body over the symphysis and sweeping the posterior arm down over the foetal chest, then turning the other shoulder back and repeating the manouver is generally readily successful.

In cases where the arms are found to be very high up perhaps aside of the head, the introduction of the hand and arm of the accoucheur aside of the foetal body sufficiently high to reach them and bring them down is difficult and very sure to result in harm to the child and in severe lacerations of the maternal parts.

Here the method of Deventer is of inestimable value. This consists in pulling the foetus directly downwards towards the floor, the patient's pelvis being well over the edge of the operating table, the arms becoming extended aside of the foetal head and resting in the yielding sacro sciatic spaces. By this means the shoulders can be brought so low that the arms become readily accessible, and can then be delivered in the usual way. Deventer even advised to continue the traction bringing the foetal body well under that of the mother and leaving the arms in position deliver by letting the occiput and the forehand to pass from under the symphysis while the face and chin rest on the posterior wall of the pelvis and perineum.

This complete Deventer manouver the writer has practiced a few times but so far only in cases where the child was rather small.

A mistake frequently made is that seen in efforts to early turn the foetal back anteriorly. This practice is based on the fear that the occiput may turn backward and delivery thus be made unnatural and difficult. It is however a practice that is both needless and harmful.

The normal mechanism of labor demands that the head shall go through the superior strait with the sagittal suture transversely, with biparietal diameter antero posteriorly and a little to one side of the promontory; also that the hips and especially the shoulders pass through the pelvic outlet in the antero posterior diameter. Turning the back anteriorly tends to result in the head becoming caught at the brim with the occipito frontal or occipito mental diameter catching in the conjugate the shortest diameter of the brim, and in the shoulders engaging in the narrow transverse diameter of the outlet. With proper management there is absolutely no danger of the occiput turning backwards in these cases. With the hips and shoulders normally delivered in the antero posterior diameter of the outlet, the fingers are introduced alongside the face and into the mouth, this is pulled downwards and backwards towards the sacrum and the head thus rotated the delivery takes place with occiput coming from under the symphysis.

In cases where the arm is already prolapsed, it is not necessary nor generally even advisable to reposit before proceeding to version and extraction. The arm is but little in the way and its prolapse is sometimes an advantage. As soon as the proper foot is reached and is being brought down, the prolapsed arm can be grasped and by upward pressure upon it used to press up the cephalic pole, thus guarding against the doubling and wedging of the foetal parts. A fillet attached to the wrist of the prolapsed arm may serve a useful purpose in again bringing down the arm at the time of the final delivery. Care must be taken to keep it aseptic. In all these cases, of which many are neglected cases with impaction and with more or less retraction of the uterus the upward pressure must be measured and cautious, otherwise there will be great danger of rupture of the lower uterine segment. Whether in some cases attempts at version are safe is a matter for the most enlightened judgment. Of course if the child is no longer living decapitation is the operation indicated.

In cases of delayed extraction due to obstruction at the brim, and with the child surely dead, the writer has found Braun's decapitating hook hooked into the orbit, a very useful means of traction upon the after coming head. Its employment in this way may avoid a cranitolomy.

The delivery of the after coming head by means of the forceps is rarely necessary. The forceps are especially useful in cases where the delay is due to an imperfectly dilated cervix. With proper management, as has been explained, these cases should not occur.

In practically all deliveries under deep anaesthesia it is the writer's practice to deliver the placenta manually immediately after the birth of the child. With proper aseptic precautions this practice need not lead to infection nor need it result in post partum haemorrhage. The thorough irrigation of the uterine cavity with a one per cent solution of Churchill's tincture of iodine is strongly recommended in all these cases where the hand or instruments have been introduced into the uterus. The syringe nozzle or tip should be brought well up to the fundus, where its presence can be recognized by the hand over the abdomen.

---

**Cerebral Tuberculosis.** Lenoble, Le Prog. Med., Apr. 20, reports the case of a tuberculous patient with left hemiplegia. Necropsy demonstrated cerebral softening, tuberculosis being demonstrated by inoculation of a guinea pig.

---

**Shell Turners' Pustules.** Thiberge, Le Prog. Med., Mch. 30, states that the impure oil used in lathe work collects iron dust and, lodging on the skin, favors the development of pustules. The iron may cause a pigmentation.

---

**Extraction of 472 Projectiles Under X-rays.** Chauvel and Loiseleur have had this experience. Even old, encystaed projectiles yielded cultures of staphylococcus.

---

**Vesico-Rectal Fistulae.** Tuffier, Le Prog. Med., Mch. 9, states that he has seen 18 out of 20 cases heal spontaneously after cystotomy.

